# Gold-based nanoplatform for a rapid lateral flow immunochromatographic test assay for gluten detection

**DOI:** 10.1186/s42490-022-00062-2

**Published:** 2022-05-20

**Authors:** Arefe Momeni, Mohammad Rostami-Nejad, Reza Salarian, Mohammad Rabiee, Elham Aghamohammadi, Mohammad Reza Zali, Navid Rabiee, Franklin R. Tay, Pooyan Makvandi

**Affiliations:** 1 Biomaterials Group, Department of Biomedical Engineering, Amirkabir University of Technology, Tehran, Iran; 2 Gastroenterology and Liver Diseases Research Center, Research Institute for Gastroenterology and Liver Diseases, Shahid Beheshti University of Medical Sciences, Tehran, 1985714711 Iran; 3 Biomedical Engineering Department, Maziar University, Royan, Iran; 4 Basic and Molecular Epidemiology of Gastrointestinal Disorders Research Center, Research Institute for Gastroenterology and Liver Diseases, Shahid Beheshti University of Medical Sciences, Tehran, Iran; 5 School of Engineering, Macquarie University, Sydney, New South Wales 2109 Australia; 6 Department of Physics, Sharif University of Technology, Tehran, Iran; 7 The Graduate School, Augusta University, Augusta, GA 30912 USA; 8 Istituto Italiano di Tecnologia, Centre for Materials Interfaces, viale Rinaldo Piaggio 34, 56025 Pontedera, Pisa, Italy

**Keywords:** AuNPs, food allergen, gluten, LFTS, rapid test, raw materials

## Abstract

**Background:**

Gluten, a food allergen, is available in foods derived from wheat, rye and barley. It damages the small intestine and causes celiac disease. Herein, we designed a rapid immunochromatographic lateral flow test assay for detecting the gluten contents of raw materials. In this rapid test, the presence of gluten was screened through the capturing of gliadin (a toxic component of gluten) by two identical gliadin monoclonal antibodies. One of the antibodies was immobilized on the membrane in the test zone as a capture reagent. The other antibody was labeled with gold nanoparticles (AuNPs) as a detector reagent.

**Results:**

Gold nanoparticles with a size of about 20 nm were synthesized and conjugated to the gliadin monoclonal antibodies. The detection limit of the experimental assay was 20 ppm and positive results were visualized after 15 min using only 40 μL of the extracted sample for each test. Analysis of different flour samples identified the best sensitivity and specificity of the lateral flow test strip (LFTS).

**Conclusion:**

The experimental LFTS is an easy-to-use and rapid method for the screening of gluten level in raw materials. The LFTS may be employed to ensure the safety of foods.

## Introduction

Food allergens can trigger immune responses and result in adverse clinical implications. They are considered a serious problem in contemporary healthcare [1]. Gliadin is a glycoprotein derived from gluten that is found in foods derived from wheat [2], rye [3], and barley [4]. Gliadin is not fully digested in the gastrointestinal tract. It damages the small intestine and causes celiac disease [5]. The major toxic component of gliadin is 33-mer peptide from alpha 2-gliadin that contains proline and glutamine amino acids residues. This peptide has frequently been described as the most important celiac disease-immunogenic sequence in gluten. The most commonly available therapy for celiac disease is a severe life-long gluten-free diet and/or the consumption of foods with a "gluten-free" label. Based on the adopted Codex standard 118-1979 by the U.S. Food and Drug Administration (FR Doc. 2013-18813) and European Commission Regulation (EC 41/2009), the level of gluten in gluten-free foodstuffs should not exceed 20 parts per million (ppm). Accordingly, monitoring of the gluten level in the labeled products is important to ensure the safety of consumer food products. Many analytical methods including polymerase chain reaction (PCR) [6], high-performance liquid chromatography (HPLC ) [7], liquid chromatography-mass spectroscopy/mass spectroscopy (LC-MS/MS ) [8], microarrays [9], immunosensors [10], Aptasensor [11], matrix-assisted laser desorption/ionization time-of-flight mass spectrometry (MALDI-TOF MS) [12] and near-infrared (NIR) spectroscopy [13] have been used experimentally for the analysis of the gluten level in non-processed and processed foods. Enzyme-linked immunosorbent assays (ELISA) and the lateral-flow assays are two common methods used for quantitative and semi-quantitative or qualitative analysis of the gluten contents in foods [14–17]. The main advantages of lateral flow immunoassay-based methods over very accurate methods like ELISA, PCR, MALDI-TOF MS and HPLC for detecting gluten in foods is their short detection time, ease of use and the capability of on-site detection without the need for expensive equipment and specialized personnel [18–21]. By applying knowledge acquired from chemistry and nanotechnology, new approaches in developing a fast, cost-effective and reliable method for gluten detection have emerged. In particular, recent interests in gluten detection have been focused on the use of inorganic nanoparticles with adjustable optical properties.

Three types of labels are employed in immunochromatographic systems. These labels included colored nanoparticles (e.g. gold, carbon and selenium nanoparticles), magnetic nanoparticles (e.g. silver nanoparticles) and luminescent (e.g. up-conversion phosphors and quantum dots). Among these labels, colloidal gold nanoparticles (AuNPs) have acceptable biocompatibility. They possess unique optical properties in the presence of different analytes, along with ease of production, conjugation and detection. Hence, AuNPs have been extensively investigated as potential labels in lateral flow immunoassays [21–28]. Lateral flow immunoassay (LFIA) is based on a paper-based biosensor and is considered as a point-of-care (POC) approach [20, 29]. This immunoassay is also known as immunochromatographic (lateral flow) assay, test strip, rapid diagnostic test, immune-gold colloid immunoassay (IG) or fluorescent quenching LFA (FQLFA) strips [30–33]. To date, lateral flow immunoassay has been applied in different fields such as food safety, clinical agriculture and environmental monitoring. Several LFIA kits for gluten detection are commercially available. They are marketed as EZ gluten®, Gluten Rapid Kit, reveal 3-D for Gluten, AgraStrip®LFD Gluten G12 and AgraStrip®LFD Gluten [34–36].

**Fig. 1:**
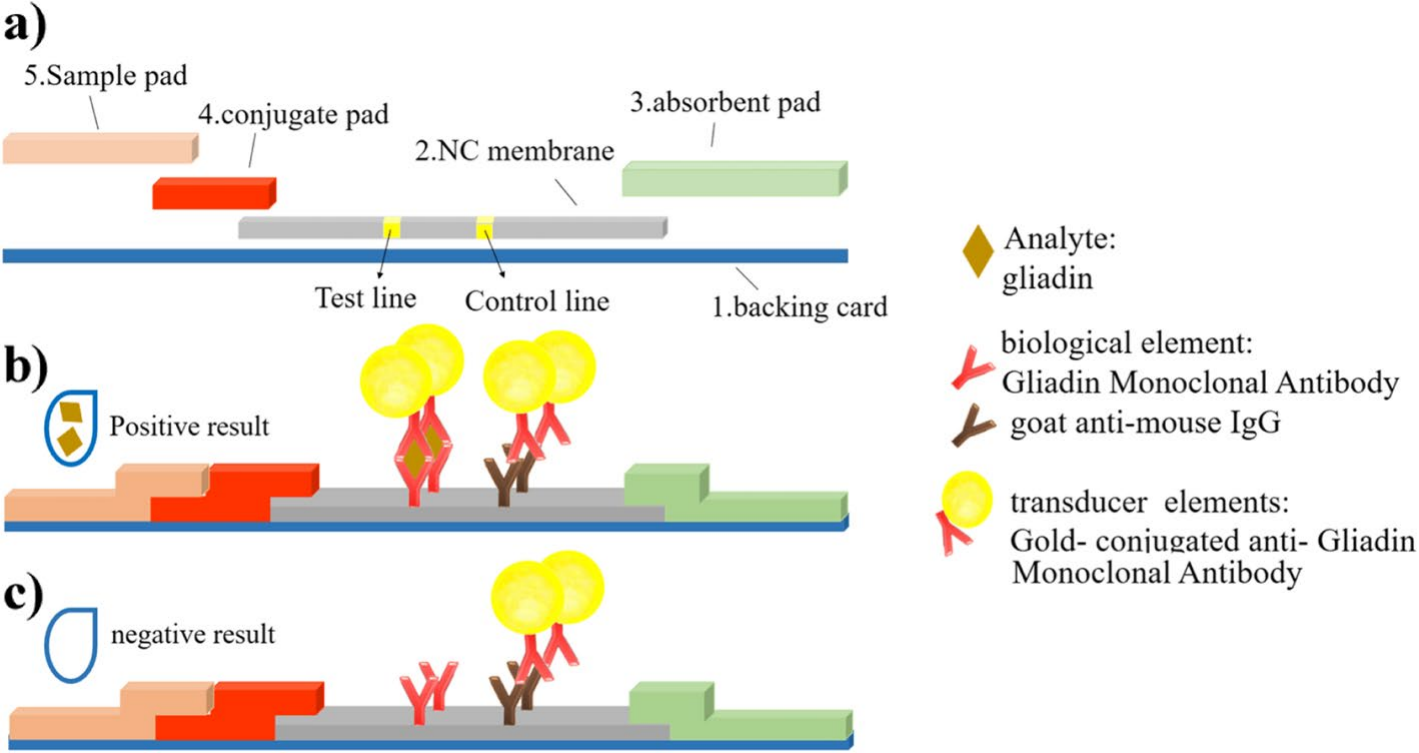
Schematic of structure and function of the lateral flow test strip. (**a**) The major components of the test strip. (**b**) Positive result and (**c**) negative result after addition of the food sample

Herein, we developed a sensitive and specific sandwich-like lateral flow test strip for gluten detection in non-processed foods, with a detection limit of 20 ppm (the Codex standard). Apart from the availability of commercial gluten detection kits with lower detection limits and more accurate methods such as ELISA, our goal is to design the simplest gluten detection kit for accurate detection of 20 ppm of gluten in food substances. This test generates results in just 15 minutes without the need for additional equipment. The test may be used by unskilled personnel of all ages and in remote areas that do not have access to advanced medical laboratories. The new test strip utilized the Gliadin Monoclonal Antibody that specifically targets the immune-dominant sequence PQPQLPY in the gliadin peptide. The augmented specificity of the novel lateral flow test strip was attributed to its higher affinity for the antigen. The lateral flow test strip (LFTS) was shown to be a user-friendly method for the rapid detection of gluten in a short time. A schematic of the structure and function of the LFTS is shown in Fig. 1a. A positive result is visualized by the appearance of two lines, the test and control lines, on the test strip (Fig. 1b), in the presence of 20 or more ppm of gluten in a food sample. The presence of only the control line on the test strip is indicative of a negative result, in the presence of less than 20 ppm of gluten in a food sample (Fig. 1c).

## Results

### Characterization of AuNPs

**Fig. 2:**
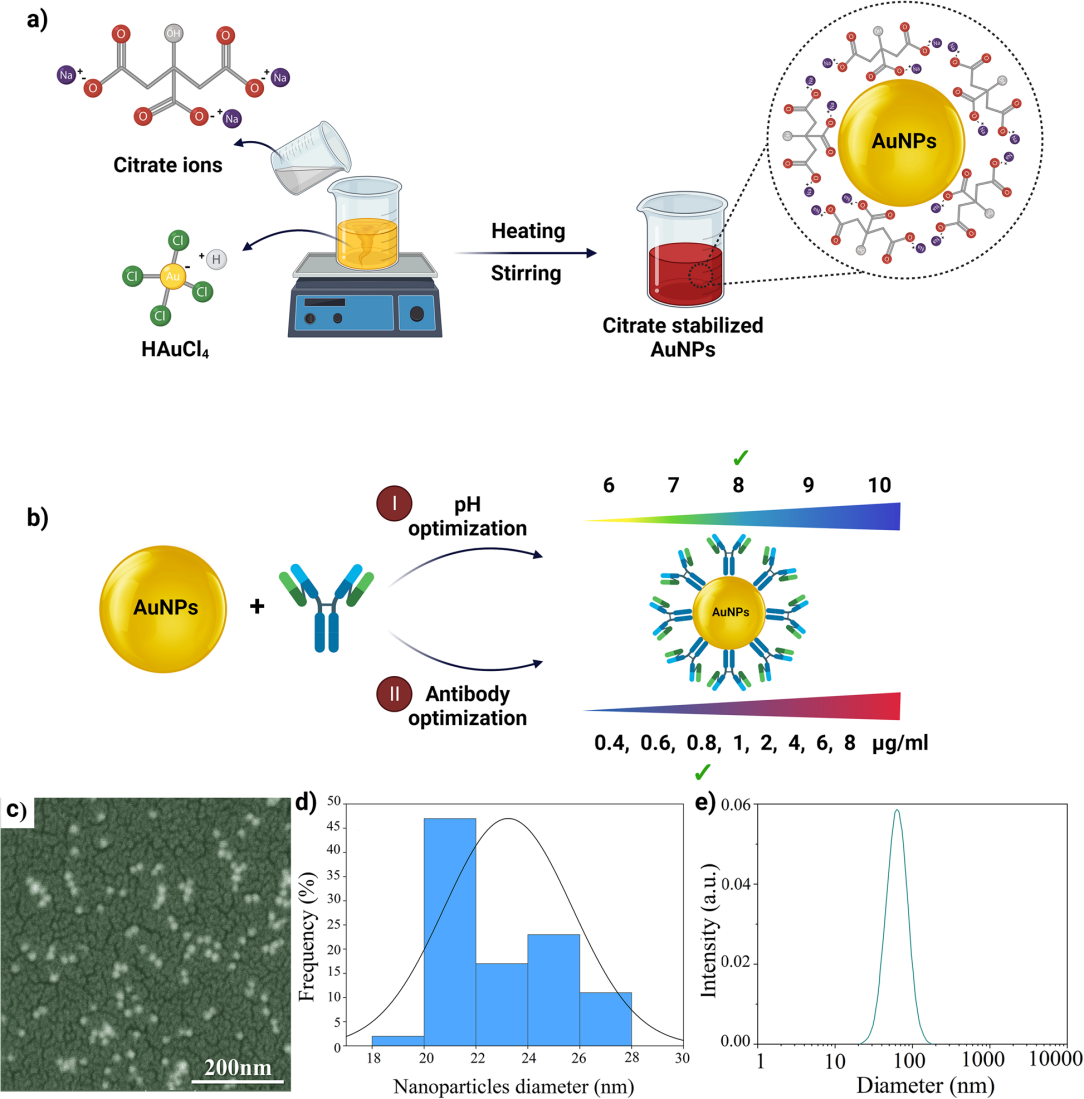
Synthesis and characterization of AuNPs with different methods (**a** and **b**). Schematic of the fabrication method. (**c**) FESEM images of the AuNPs without antibody; (**d**) Diagram of the size distribution of AuNPs based on measurement of the diameter of 800 AuNPs; (**e**) Dynamic light scattering analysis for determination of the z-average hydrodynamic diameter of the AuNPs

Gold nanoparticles were synthesized using citrate reduction in several steps that involved nucleation (reduction of HAuCl_4_ to gold atoms), growth and agglomeration of atoms into nanoclusters to approximate 20 nm in diameter (Fig. 2a and b). The size distribution and the average diameter of the AuNPs were determined based on the field emission scanning electron microscopy (FESEM) and ultraviolet-visible (UV-VIS) light spectrophotometry (Fig. 2c and d, blue line). The hydrodynamic diameters of the AuNPs were measured using dynamic light scattering (Fig. 2e). Based on the FESEM images (Fig. 2a), the AuNPs were spherical and had a uniform size distribution of about 20 nm (analyzed with ImageJ software, Fig. 2b). The hydrodynamic diameter and polydispersity index of the nanoparticles were 23 nm and 0_._1, respectively (Fig. 2e). The spectral results revealed a narrow absorption peak at 523 nm, which is indicative of the presence of uniform, spherical AuNPs.

### Characterization of antibody-AuNPs conjugates

#### pH optimization

**Fig. 3:**
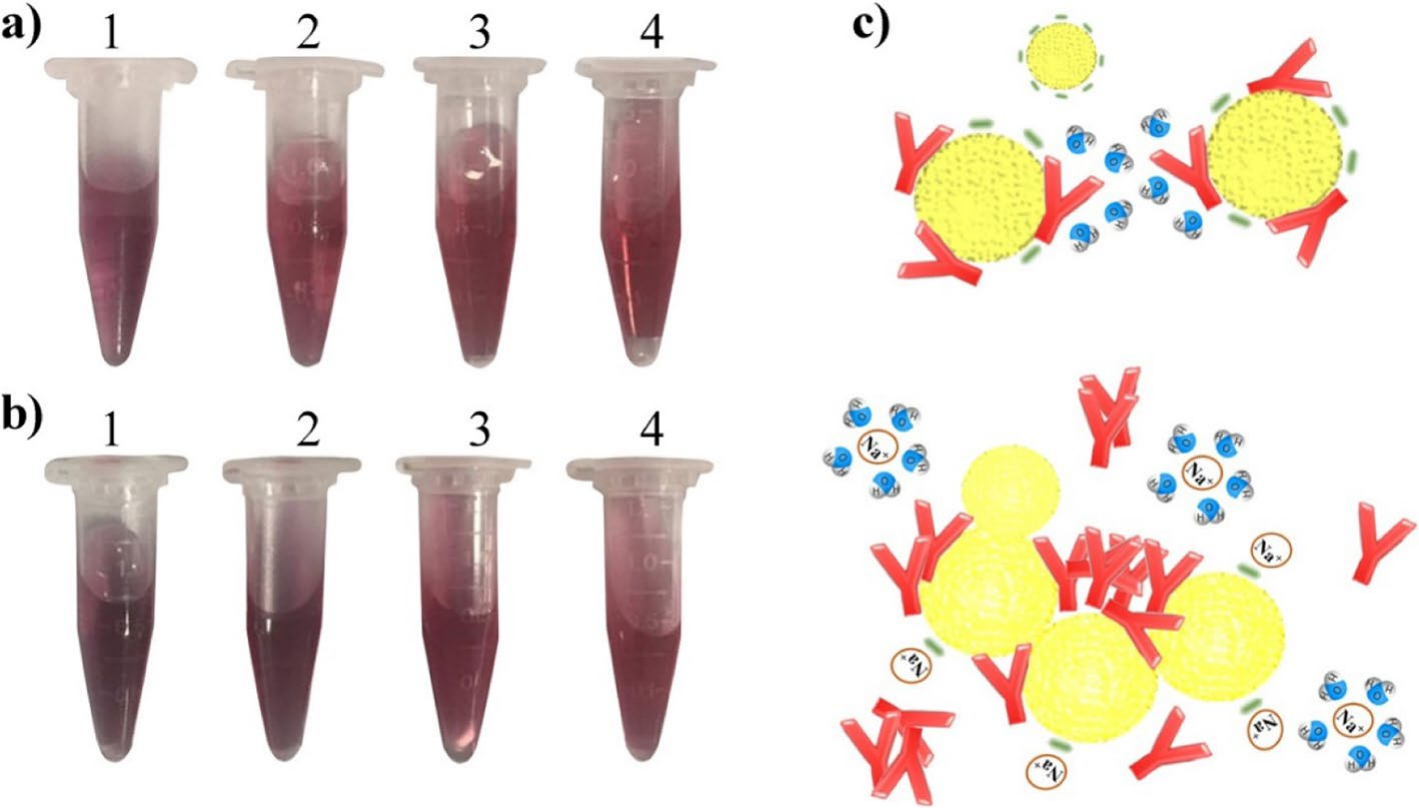
Optimization of pH for conjugation. Color changes AuNPs at different pH values (1: 6, 2: 7, 3: 8 and 4: 9) (**a**) without antibody (**b**) with 1 μg/mL antibodies after adding NaCl. (**c**) Schematic of the aggregation of AuNPs via protein interaction in presence of NaCl

To determine the optimum pH for conjugation, the color change of the AuNPs at different pH values was recorded before and after the addition of antibodies, in the presence of 1_._5 M NaCl. Fig. 3a shows the color change of the AuNP solution from red to purple after the addition of NaCl into the solution at a low pH value (pH = 6-7), which resulted in the aggregation of the AuNPs. At pH values above 7.0 (pH = 8 or 9), no red-to-purple color change was observed; the color of the solution changed to dark red instead. The color changes of the solution became more visible after the addition of a designated concentration (see below) of antibodies to each Eppendorf tube, in the presence of NaCl (Fig. 3b). The most optimal pH value for the conjugation procedure was found to be 8. The schematic of the mechanism is shown in Fig. 3c.

#### Optimal concentration for conjugation

**Fig. 4:**
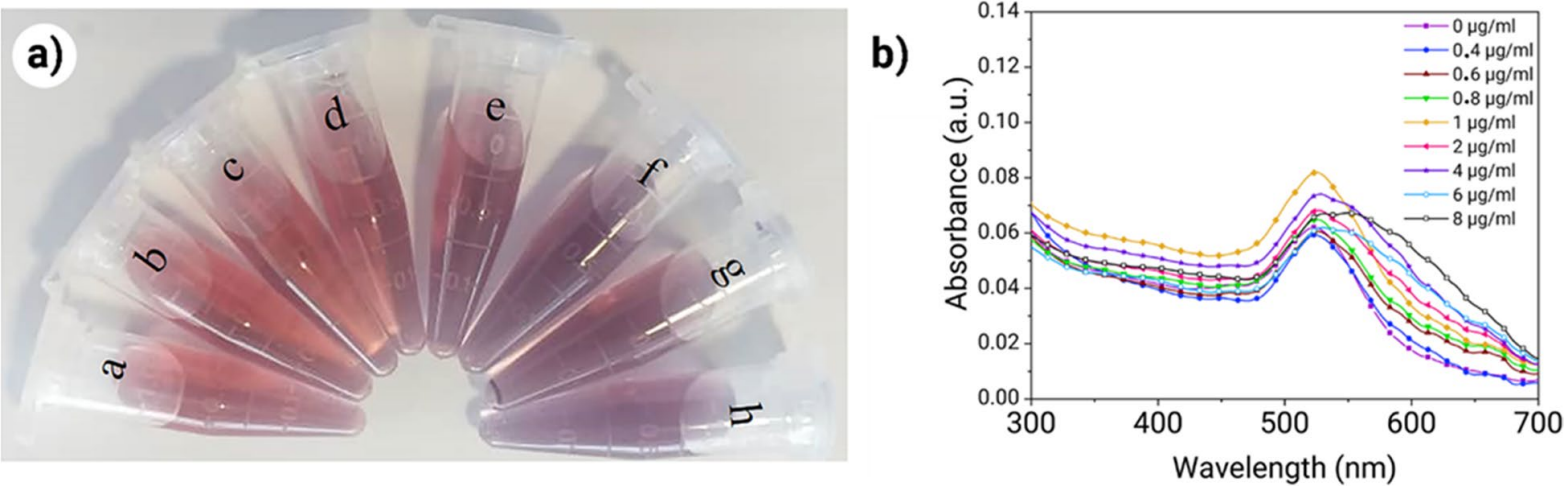
Optimization of antibody concentration for conjugation. (**a**) Color changes of the AuNPs with different antibody concentrations (0.4, 0.6, 0.8, 1, 2, 4, 6, 8 μg/mL) in the presence of NaClchanges were observed by the naked eye to determination optimal pH). (**b**) UV–Vis spectra of AuNPs with various antibody concentrations in presence of NaCl

At optimal pH, the optimal concentration of antibody was determined to be 1 μg/mL, which was the highest antibody concentration that maintained stable, conjugated AuNPs in solution. These results were obtained by observing the color change of AuNPs containing different antibody concentrations in presence of NaCl. Fig. 4a shows pH-adjusted gold solutions containing different antibody concentrations (0-8 μg/mL) in the presence of NaCl. As shown in Fig. 4a, when the concentration of antibody increased from 0 to 0_._8 μg/mL, only a slight color change was observed. Conversely, at concentrations above 1 μg/mL, the color of the AuNPs changed from deep red to purple, which was indicative of the aggregation and precipitation of the AuNPs. To validate the result, the UV–VIS absorption spectra of the synthesized AuNPs were measured and compared with each other (Fig. 4b). With the use of 1 μg/mL of antibody, the absorption peak at 523 nm increased and shifted toward longer wavelengths. The feature was indicative of the interaction of the antibodies with the surface of the AuNPs.

**Fig. 5:**
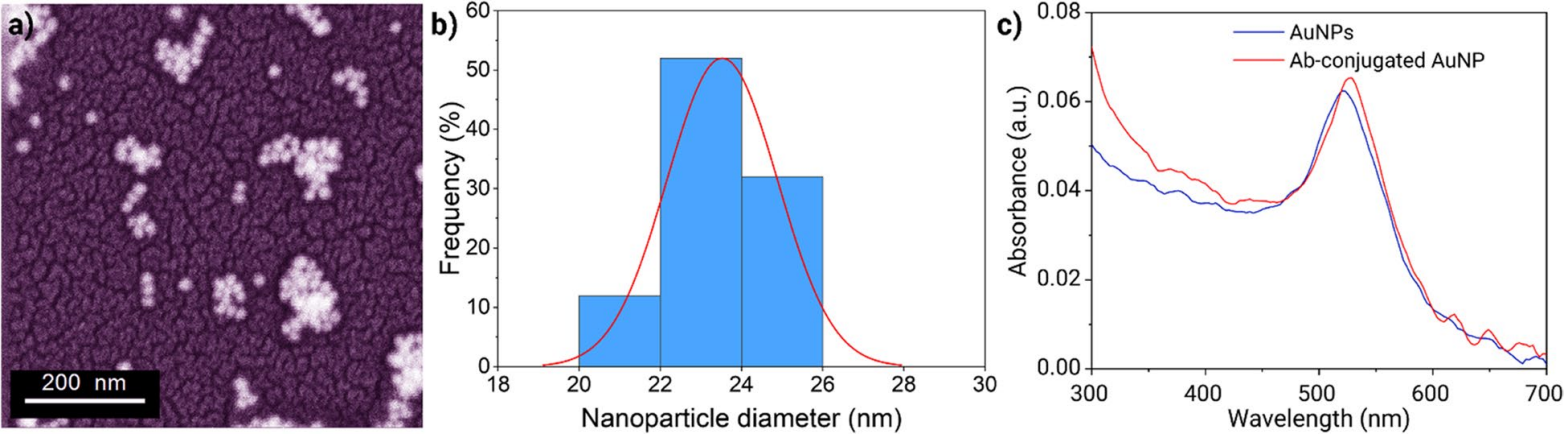
(**a** and **b**) FESEM images of antibody-conjugated AuNPs and (**c**) UV–Vis absorbance spectra of the AuNPs before (blue line) and after (red line) antibody conjugation, using the pre-determined optimal conditions for binding of antibodies to the AuNPs

Based on the aforementioned results, antibody conjugation to the AuNPs was performed at 1 μg/mL and pH 8. Fig. 5a and b shows the FESEM image of the antibody-labeled AuNPs. Binding of the antibodies to AuNPs resulted in a slight increase in the size of the AuNPs. Clustering of the labeled AuNPs on the substrate in FESEM image was ttributed to the change in surface energy of the nanoparticles after conjugation. Analysis of thesize of the antibody-labeled AuNPs in the FESEM images was performed using the ImageJ software (National Institute of Health, Bethesda, MD, USA). Most of the nanoparticles increased in size, up to 24 nm, due to the presence of adsorbed proteins on the surface of the nanoparticles; their average diameter was ~23_._5nm. The absorption spectra of the AuNPs (Fig. 5c, red line) were used to compare the status of the nanoparticles before and after conjugation at optimal conditions. The absorption peak position of the conjugated AuNPs shifted from 520-523 nm to 526-529 nm and the intensity of absorption increased at 523 nm.

#### Sensitivity of the LFTS

**Fig. 6:**
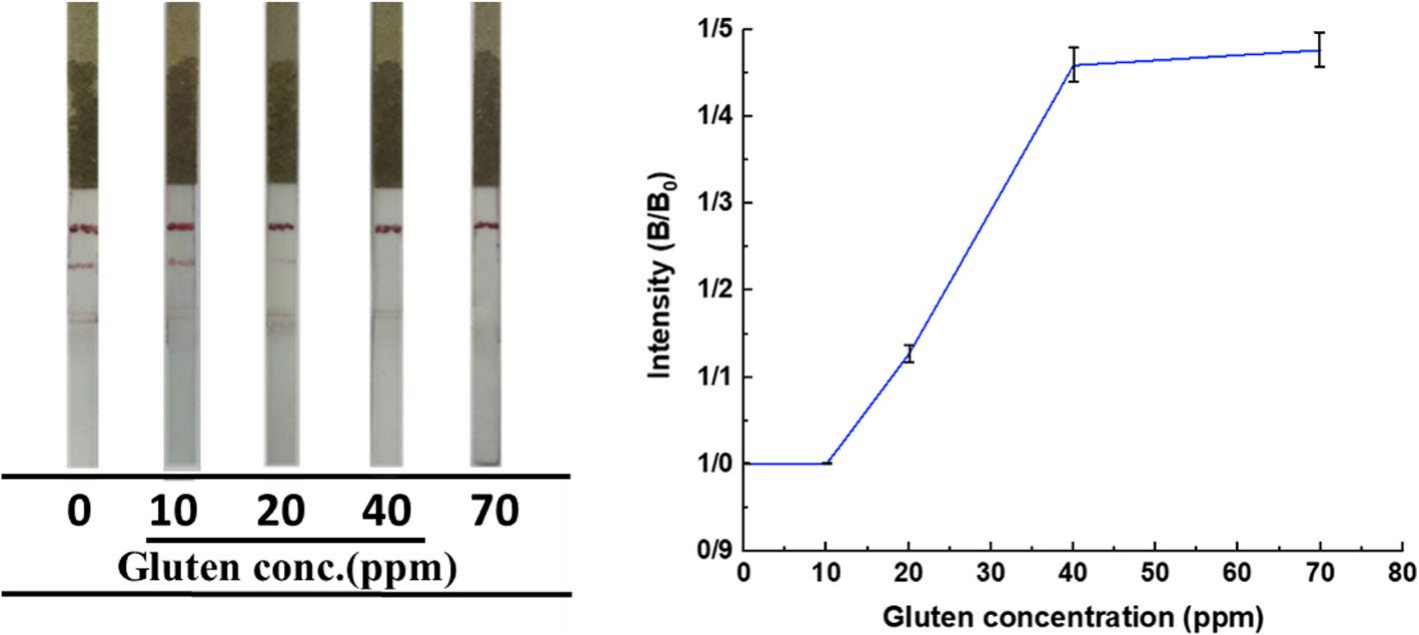
Sensitivity results of LFTS at different concentrations of gluten-containing samples

To determine the sensitivity of the LFTS, four different concentrations of gluten (0, 10, 20, 40, 70 ppm) were examined on the LFTS in triplicate. As shown in Fig. 6, a pink color appeared along the test line as the concentration of gluten increased to 20 ppm. The color intensity of the test lines increased with the addition of 40 ppm and 70 ppm gluten. The test line disappeared when the gluten concentration was below 20 ppm. Therefore, the visual detection limit of the LFTS was 20 ppm. The time required to perform the test was within 15 min. For semi-quantitative detection, the intensity of the test lines was evaluated using Image J software. A standard curve was plotted based on the percentage of the intensity of the test line spiked sample (B) to the intensity of the test line of the blank sample (B_0_) against the gluten concentration.

#### Specificity of the LFTS

**Fig 7:**
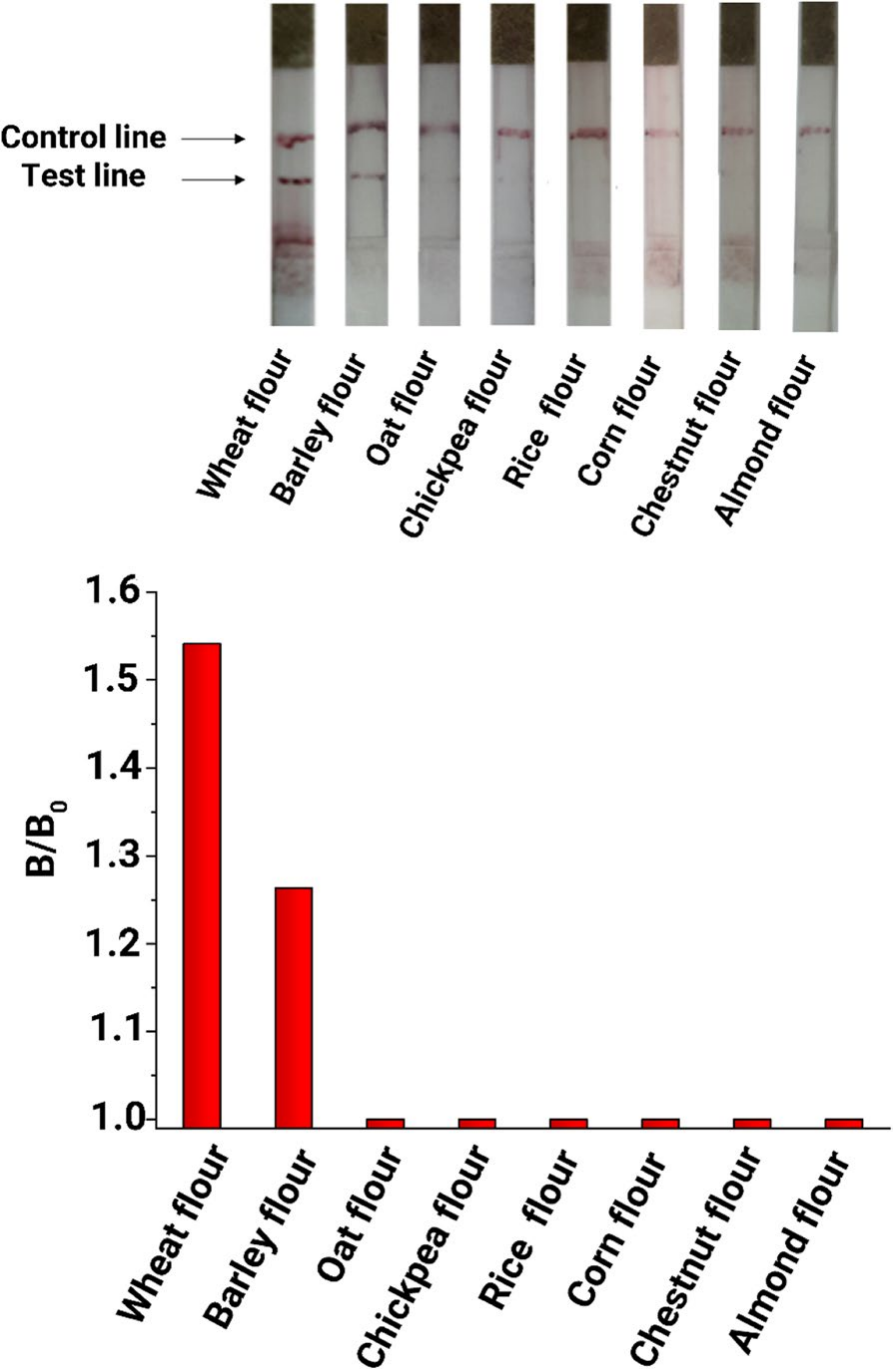
Specificity results of LFTS with different flour samples.

Different flour samples derived from wheat, oat, corn, barley, rice, chickpea, chestnut and almond flour were evaluated for examination of the specificity of the LFTS. No red line was observed on the test line except for the gluten-containing wheat and barley flour samples. In addition, a red band was formed for wheat flour sample as early as 2 min. This is because of the high concentration of gluten in the wheat flour sample (Fig. 7). Each sample was evaluated 15 times with the LFTS under the same experimental conditions. The results showed that only three of the 120 samples had false results ( barley flour : False- negative test result, rice and corn flour : False-positive test result). This was indicative of the high specificity of the experimental LFTS.

## Discussion

In the present study, an experimental lateral flow assay was developed for the detection of gluten in commercial non-processed foods. Because the extraction method of gluten from raw and processed food matrices is different, the development of a single assay for both raw and processed food materials will affect the accuracy of the assay. Accordingly, the present assay was developed only for non-processed foods. Other lateral flow assays such as dipsticks have been reported by Yin et al. [17]. However, in a lateral flow dipstick test, the sample is not applied directly on the strip and requires an additional incubation step [37, 38].

The authors synthesized AuNPs with an approximate diameter of 20 nm as the label for the LFTS. The reason for choosing AuNPs as the label was their biocompatibility, high surface-to-volume ratio, ease of synthesis, considerable optical properties and chemical stability. Gold nanoparticles of this dimension were produced because of the ease of passage of nanoparticles through the nitrocellulose membrane pores and reduced background noise. According to the literature [39, 40], AuNPs with diameters larger than 30 nm become elliptical with polydisperse size distributions. These features adversely affect the control and reproducibility of the conjugation process. The labeling efficiency or ability of analyte detection by the AuNP-conjugated antibodies decreased with increasing AuNP dimensions. This could be attributed to bad penetration, steric hindrance or repulsion forces.

Successful conjugation is a critical step in lateral flow immunoassay. Stable conjugation helps to minimize nonspecific binding and false-positive results. Hence, it was necessary to optimize the conditions (i.e., pH of the AuNP solution, antibody concentration, NaCl concentration and temperature) for the preparation of a stable conjugate. Gold nanoparticles are stable in solution because of the balance between repulsive forces that are generated between the surface of the AuNP surface and intraparticle van der Waals attractive forces. The color of the AuNP solution was red when the AuNPs were stable. Nevertheless, parameters such as pH and electrolyte concentration could alter the negative charge on the AuNP surface and affect the intraparticle repulsive force. These alterations resulted in aggregation of the nanoparticles, which was accompanied by a color change of the nanoparticle-containing solution. In the present study, the optimum pH for the formation of stable conjugation was determined to be 8. The AuNPs were more susceptible to aggregation because of the protonation of the carboxyl group of citrate ions on surface nanoparticles at lower pH values. Weakening of the intraparticle repulsive force resulted in aggregation of the AuNPs upon the addition of an electrolyte. The AuNPs were more stable at pH > 7 due to the greater intraparticle repulsive forces that resulted from deprotonation of citrate ions [41–43].

At low pH, the AuNPs became less table in the presence of antibodies. Nanoparticles aggregation occurred via protein-protein interaction for several reasons. Firstly, antibodies are likely to unfold under inappropriate pH condition (pH ≤ 7). Secondly, the presence of a salt not only destabilizes the AuNPs but also increases the tendency of the antibodies to attract one another and aggregate. When a salt is added to a protein-containing AuNP solution, water molecules surround the Na^+^ ions instead of the protein molecules. As a result, aggregation of nanoparticles occurs by the attraction of the antibodies to each other via their hydrophobic zones (Fig. 3c). Gold nanoparticles demonstrate local surface plasmon resonance absorption in the visible light region. Such an effect depends on the size, shape, aggregation state and local refractive index of the surrounding medium. The localized surface plasmon peak of the AuNPs is altered after bonding of the antibodies [44, 45]. In Fig. 4b reduction in absorption intensity occurred at antibody concentrations less than 0.8 μg/mL. This could be attributed to the instability of the AuNPs. The absorption peak at 523 nm increased and shifted toward a longer wavelength at antibody concentrations of 0.8 μg/mL and 1 μg/mL. The redshift of the absorption peak could be attributed to changes in the local refractive index of the AuNPs [46], which could happen upon the attachment of antibodies to the nanoparticles.

When the antibody concentration was higher than 1 μg/mL, the absorption peaks became broadened with several peaks appeared at longer wavelengths (Fig. 4b). This could be caused by the formation of AuNP aggregates in solution. According to the literature [47], aggregation of AuNPs is dependent on the protein concentration. At low antibody concentration (i.e. 0.4 to 0.8 μg/mL), the number of AuNPs was more than the number of antibodies and small aggregates formed via bridging of antibodies between the AuNPs. When antibody concentration reached 1 μg/mL, the solution color began to change from dark red to purple as a result of the formation of large aggregates in the solution. Although the surface of the AuNPs are covered by antibodies, there were still locations where additional antibodies may be attached in a time-dependent manner. Addition of NaCl reduced the interaction time of antibodies with nanoparticles and other antibodies. Conversely, addition of a blocking agent such as bovine serum albumin shortly after the incubation of the antibodies with AuNPs prevents the nanoaprticles from aggregating due to negative net charges at pH 8 and the hydrophilicity of bovine serum albumin. When the antibody concentration was beyond 1 μg/mL, the solution turned dark purple and eventually became colorless. This could be attributed to the desorption of antibodies from the gold nanoparticle surface, with the formation of large aggregates of antibodies on AuNP surface that eventually induced aggregation of the nanoparticles via their own hydrophobic regions.

Table 1 compares the present LFTS assay with other assays. In the present work, the LFTS sensitivity was optimized to 20 ppm to enable facile application by untrained individuals on-site without need to check their test results with standard charts or a color scale card, or the use of an image analyzer for concentrations lower than 20 ppm. This impermissible gluten content (< 20 ppm) is determined based on the observation of colored lines in the test and control zones by the naked eye. Based on the literature [54–56], the sensitivity of this LFTS is in an appropriate range. Evaluation of the food samples with the use of 120 LFTS demonstrated the high specificity of the present experimental LFTS. The method may have practical clinical applications as well, as a user-friendly, rapid and low-cost diagnostic test for monitoring gluten intae in patients with celiac disease.

**Table 1:** Comparison of the present LFTS assay with other lateral flow assay

Type of assay	Detection limit of gluten (ppm)	Type of antibody	Ref.
Present experimental LFTS	20	Gliadin Monoclonal Antibody (14D5)	-
Barcode-style LFA dipstick	50 - 100	Anti-gliadin IgY	[17]
2B9- based lateral flow device	2	Monoclonal antibody against deamidated gluten (2B9)	[48]
RIDA®QUICK Gliadin dipstick	2.5	R5 (Mendez) monoclonal antibody	[49]
GlutenTox Sticks	3	G12 monoclonal antibody	[50]
3M™ Gluten Protein Rapid Kit	5.0	Polyclonal antibody	[51]
Reveal® 3-D for Gluten	10	401.21 monoclonal antibody (Skerritt)	[52]
EZ gluten®	10	401.21 monoclonal antibody (Skerritt)	[53]
Proteon Gluten Express Dipstick	3	G12 monoclonal antibody	[53]

## Conclusions

In the present work, a paper-based lateral flow immunoassay was developed for monitoring the level of gluten in non-processed food. The method may be used for rapid and accurate detection of gluten in 15 min or less. The limit of detection of this assay was 20 ppm, which is equal to adopted threshold based on the Codex Standard 118-1979. The amount of antibodies at the test line, the amount of the conjugated particles on the conjugate pad, the size of the AuNPs, the dimensions of the strip and the position of the test and control lines affect the performance and sensitivity of the developed assay. Here, the highest concentration of antibodies that enabled the AuNPs to remain stable in the presence of NaCl was chosen as optimal antibody concentration. The activity of the conjugates was confirmed based on proper performance of the LFTS. The experimental LFTS with high sensitivity and specificity may be used as an alternative to other multistage and more time-consuming methods such as ELISA for detection of gluten in non-processed foods.

## Methods

Hydrogen tetrachloroauratetrihydrate (HAuCl_4_·3H_2_O) was purchased from Millipore Sigma (Shanghai, China). Nitrocellulose membranes (Hi-Flow plus 120, Merck Millipore), sample pad (cellulose fiber, Merck Millipore), conjugate pad (cellulose fiber, Merck Millipore), absorbent pads, adhesive backing cards and goat anti-mouse IgG were obtained from Rojan Azma Co (Tehran, Iran). Trisodium citrate dehydrate, bovine serum albumin, Tween-20, phosphate buffer solution, PEG20000, glucose and 0.22 μm filters were purchased from Merck Millipore (Burlington, MA, USA). Gliadin Monoclonal Antibody (14D5) was purchased from Thermo Fisher Scientific (Waltham, MA, USA).

### Synthesis of AuNPs

To obtain the monodisperse AuNPs solution, all glasswares and magnets were washed with aqua regia (3 HCl: HNO_3_ vol/vol) and rinsed in distilled water. All solutions were filtered through a 0.22 μm syringe filter. A silicone oil bath was used for uniform heating. The AuNP solutions were synthesized by citrate reduction method [57]. Briefly, 3 mL of 1% trisodium citrate solution was added to 95 mL of near-boiling solution of 0.01% tetrachloroauric acid under vigorous stirring. After 20 min., the color of the solution turned to red and after stirring for another 10 min., the solution was stored under refrigeration at 4 °C in the dark. The synthesized AuNPs were characterized by field emission scanning electron microscopy (FESEM; MIRA3 Tescan, Kohoutovice, Caech Republic), Nano-Drop ND-1000 Spectrophotometer (Thermo Fisher Scientific Inc) and dynamic light scattering (DLS, VASCO2, Cordouan Technologies, Pessac, France).

### Preparation of AuNP-conjugated antibody

Binding of antibodies to the surface of AuNPs was performed by passive adsorption [58, 59]. Despite the simplicity of this method, the control of optimal conjugation conditions such as pH and antibody concentration is important for preparing a stable conjugate.

To determine the optimized pH, the pH value of the AuNPs was adjusted to different levels (6, 7, 8, 9, 10) by addition of 0.2 M K_2_CO_3_. Then, 100 μL of gliadin antibody solution (1 *μ*g/mL) was added to Eppendorf tubles containing 1 mL of AuNPs, under rotating for 30 min. Subsequently, 40 *μ*L of NaCl (1.5 M) was added to each tube and incubated for 15 min, with careful monitoring of the color change. Optimal pH was selected based on the color change of the solution.

To determine the optimal antibody concentration, 100 μL of different concentrations of antibody (0_._4 μg/mL, 0_._8 μg/mL, 1 μg/mL, 2 μg/mL, 4 μg/mL, 6 μg/mL and 8 μg/mL) was added to 1mL of AuNPs at the optimized pH and incubated for 30 min. Then, 40 μl of NaCl (1.5 M) was added to the solutions and incubated for another 15 min. The absorbance of solutions before and after conjugation was measured by UV-VIS spectrophotometry.

For conjugation of antibodies to the AuNPs*,* the optimized concentration of antibodies was added dropwise to the pH-adjusted AuNPs and incubated at room temperature for 1 h. Bovine serum albumin (10%) was added to block the remaining surface of the AuNPs. The mixture was centrifuged (10000 rpm for 30 min at 4 °C) to remove supernatant. Centrifugation was repeated twice. The obtained pellet was re-suspended in 1% bovine serum albumin and stored at 4 °C until use.

### Preparation of LFTS

The test strip consisted of a sample pad, conjugate pad, nitrocellulose membrane, absorbent pad and adhesive backing cards. These components were cut with dimensions 5 mm × 20 mm, 5 mm × 5 mm, 5 mm × 25 mm, 5 mm × 30 mm, 5 mm × 75 mm, respectively. After determining the optimal antibody concentration, Gliadin Monoclonal Antibody (14D5) (60 μg/mL) and goat anti-mouse IgG (100 μg/mL) were dispensed at a distance of 6 mm from each other onto the nitrocellulose membrane to form the test and control lines, respectively. The pH level and the proportion of the components in the blocking buffer was optimized during pre-treatment of the sample pad. The sample pad was soaked in phosphate-buffered saline containing 5 % (w/v) bovine serum albumin, 0.5 % Tween 20, 5 % polyethylene glycol and 0.05 % (w/v) NaN_3_ for 30 min. The treated sample pad was rinsed with phosphate-buffered saline and dried overnight at 37°*C*. The conjugate pad was made by soaking of the pad in the conjugate solution and incubated overnight at 37°*C*. All pads were ultimately laminated according to Fig. 1a.

### Sample preparation and test procedures

Gliadin was extracted from the flour samples as follows. One gram of flour was stirred with 10 mL 60% (v/v) ethanol solution to create a homogeneous solution. The solution was centrifuged at 6000 rpm for 10 min at room temperature and the supernatant was diluted with phosphate-buffered saline (pH 7.4; 1:20 v/v).

Testing was performed by the application of 100 μL of sample solution on the sample pad. The latter was moved to contact the conjugate pad. In the presence of the analyte, a complex was formed between gliadin and the AuNP-labeled gliadin antibody at the conjugate pad. The produced complex and additional AuNP-labeled antibodies moved through the membrane by capillary effect and were subsequently captured by immobilized antibodies on the test line (detection zone) and the control line, with the appearance of two visible lines on the membrane. In the absence of the analyte, only the control line was visible. Excess solution was absorbed by the absorbent pad.

### Specificity and sensitivity of LFTS

Gluten from the designated food samples was extracted with 60% (v/v) ethanol to produce standard solutions with final gluten concentrations of 0, 10, 20, 40 and 70 ppm. The supernatant was filtered through filter paper with 0.45 μm pore size. The extracted sample was diluted with phosphate-buffered saline and used for the determination of the sensitivity of the LFTS. The images of the strips were captured using a camera and analyzed using ImageJ software. The specificity of the assay was examined using the eight other flour samples (wheat, oat, corn, barley, rice, chickpea, chestnut, almond flour). Gluten was extracted from these good samples as mentioned above and the color intensity of the test lines was determined using ImageJ software.

## Data Availability

The datasets used and/or analysed during the current study available from the corresponding author on reasonable request.

## Abbreviations

AuNPs: gold nanoparticles
LFIA: Lateral flow immunoassay
POC: point-of-care
LFTS: lateral flow test strip
ppm: parts per million
PCR: polymerase chain reaction
HPLC: high-performance liquid chromatography
LC-MS/MS: liquid chromatography
MALDI-TOF MS: matrix-assisted laser desorption/ionization time-of-flight mass spectrometry
NIR: near-infrared
ELISA: enzyme-linked immunosorbent assays
IG: immune-gold colloid immunoassay
PBS: phosphate-buffered saline
BSA: buffered saline
